# Effect of Injury Patterns on the Development of Complications and Trauma-Induced Mortality in Patients Suffering Multiple Trauma

**DOI:** 10.3390/jcm12155111

**Published:** 2023-08-03

**Authors:** Nils Becker, Antonia Hammen, Felix Bläsius, Christian David Weber, Frank Hildebrand, Klemens Horst

**Affiliations:** 1 Department of Orthopedics, Trauma and Reconstructive Surgery, RWTH University Hospital Aachen, 52074 Aachen, Germany; nibecker@ukaachen.de (N.B.);; 2 Department of Psychiatry, Psychotherapy and Psychosomatics, RWTH University Hospital Aachen, 52074 Aachen, Germany

**Keywords:** polytrauma, complication, injury, trauma, mortality

## Abstract

Patients that suffer from severe multiple trauma are highly vulnerable to the development of complications that influence their outcomes. Therefore, this study aimed to evaluate the risk factors that can facilitate an early recognition of adult patients at risk. The inclusion criteria were as follows: admission to a level 1 trauma center, injury severity score (ISS) ≥ 16 (severe injury was defined by an abbreviated injury score (AIS) ≥ 3) and ≥18 years of age. Injury- and patient-associated factors were correlated with the development of four complication clusters (surgery-related, infection, thromboembolic events and organ failure) and three mortality time points (immediate (6 h after admission), early (>6 h–72 h) and late (>72 h) mortality). Statistical analysis was performed using a Chi-square, Mann–Whitney U test, Cox hazard regression analysis and binominal logistic regression analysis. In total, 383 patients with a median ISS of 24 (interquartile range (IQR) 17–27) were included. The overall mortality rate (27.4%) peaked in the early mortality group. Lactate on admission significantly correlated with immediate and early mortality. Late mortality was significantly influenced by severe head injuries in patients with a moderate ISS (ISS 16–24). In patients with a high ISS (≥25), late mortality was influenced by a higher ISS, older age and higher rates of organ failure. Complications were observed in 47.5% of all patients, with infections being seen most often. The development of complications was significantly influenced by severe extremity injuries, the duration of mechanical ventilation and length of ICU stay. Infection remains the predominant posttraumatic complication. While immediate and early mortality is mainly influenced by the severity of the initial trauma, the rates of severe head injuries influence late mortality in moderate trauma severity, while organ failure remains a relevant factor in patients with a high injury severity.

## 1. Introduction

Aside from high mortality rates at the scene of an accident or injury, the development of severe complications remains responsible for an unfavorable outcome in multiple-trauma patients [1,2]. Due to a high vulnerability to posttraumatic complications, special attention must be given to those patients at risk. Considering the huge heterogeneity among multiple-trauma patients in regard to trauma pattern, injury severity, patient-specific factors (e.g., age, co-morbidities, genetic predisposition) and applied treatment strategies, the individual risk of developing posttraumatic complications is highly variable [3,4,5,6]. Despite improvements in trauma management over the last decades that have led to significant benefits, the injury and patient variables that might predict the further clinical course at a very early stage require further investigation [7,8]. Both trauma severity and pattern are known to have an impact on the intensity of the posttraumatic immunologic response. An overwhelming immune response might negatively affect organ function and potentially lead to the development of multiorgan dysfunction syndrome (MODS), which is frequently associated with a poorer outcome [9].

Therefore, trauma severity is frequently included in prediction models. Furthermore, other parameters that are available at a very early stage after hospital admission, like the Glasgow Coma Scale (GCS), the injury mechanism but also the time of initial surgery and admission to intensive care units (ICUs), can be found in such models [10,11,12,13]. Parameters available over the further clinical course, such as ICU stay and ventilation time, have also been shown to influence the prognosis in multiple-trauma patients [14,15]. Against this background, we hypothesize that (a) severe injuries in distinct anatomical regions correlate with specific clusters of complications, and (b) the development of distinct clustered complications is a time-dependent risk factor for trauma-associated mortality.

To evaluate these hypotheses on the trauma-associated development of complications and their time-dependent influence on mortality, we enrolled this retrospective analysis of patients with severe multiple trauma in a level I trauma center, assessing the impact of injury- and patient-associated factors.

## 2. Materials and Methods

### 2.1. Inclusion and Exclusion criteria

#### 2.1.1. Inclusion Criteria and Ethics

This retrospective analysis included consecutive treated patients aged ≥ 18 years with an Injury Severity Score (ISS) ≥ 16 who were submitted to the emergency department and admitted to the ICU of the local level I trauma center between 2010 and 2014. This study was reviewed by the local ethics committee (Ethik-Kommission der RWTH Aachen, Aachen, Germany). Due to the retrospective character of this study, no formal ethics approval was required (EK 23-071).

#### 2.1.2. Exclusion Criteria

Patients that died prior to arrival at the emergency department were excluded. Also, patients aged < 18 years (n = 27) or those showing incomplete data (n = 18) were excluded from the analysis.

### 2.2. Data Collection

#### 2.2.1. Demographics

Data collection included general patient parameters (age, gender, weight and body-mass index (BMI)), information about the trauma mechanism, as reported by the emergency medical service, and all diagnoses revealed after hospital admission.

#### 2.2.2. Clinical Course

Data were collected using the hospital’s documentation system, including the admission assessment, daily documentations, operative reports and discard papers. The collected data included the duration of mechanical ventilation and ICU stay, and the duration of emergency surgery (defined as an operation within the first 24 h after admission). Furthermore, information about the development of complications and mortality was collected.

#### 2.2.3. Status at Admission and Scoring Systems

Patients were treated according to the current Advanced Trauma Life Support^®^ (ATLS^®^) and national guidelines [16] with total-body computed tomography (CT scan) when indicated. After admission, the lactate level was directly measured by venous blood gas analysis. The neurological status was classified by the Glasgow Coma Scale (GCS) [17]. The injury severity was assessed via the Abbreviated Injury Scale (AIS) [18], on the basis of all clinical and radiologically findings, with a subsequent calculation of the ISS [19].

To score physiologic parameters at admission, the Sequential Organ Failure Assessment (SOFA) score was used. Therefore, six different organ functions were evaluated, including respiratory, circulatory, hepatic, renal, coagulation and neurological parameters, as described before [20]. In each region, a maximum score of 4 points was defined and combined as the SOFA score.

### 2.3. Endpoints

Primary endpoint mortality was defined for those patients who died during the clinical course after first admission following trauma. The secondary endpoint was assessed as the development of complications.

#### 2.3.1. Mortality

In-hospital mortality was divided time-dependently into immediate (death within 6 h after admission), early (>6 h–72 h after admission) or late death (>72 h after admission). Time points were chosen to differentiate between the previously described first peak of mortality [3] and later in-hospital mortality. Subdivision after 72 h was implemented, to discriminate trauma- or patient-related factors leading to previously described rapid multiorgan failure (MOF), which is associated with a higher mortality than late-onset MOF [21] and factors, leading to a delayed posttraumatic mortality.

#### 2.3.2. Complications

Diagnosed complications were identified from the hospital documentation system as classified by the treating physician. Complications were grouped into four clusters. The cluster “infection” includes pneumonia, urinary tract infections, wound infections and sepsis (defined by the classical sepsis criteria [22]). The cluster “thromboembolism” includes patients that developed myocardial infarction, brain infarction, thrombosis or pulmonary embolism. In the cluster “surgical treatment associated (surgery)”, compartment syndrome, hematoma or seroma, wound-healing disorders, nerve damage or implant-associated complications are included. Lastly, organ dysfunctions were included in the cluster “organ failure”, assessing acute respiratory distress syndrome (ARDS) and acute kidney failure.

### 2.4. Statistics

The statistical analysis was performed using Microsoft Excel (Windows, version 16.66.1) and statistical package IBM SPSS Statistics software (SPSS 25, IBM Inc., Armonk, NY, USA). Normality was assessed using a Shapiro–Wilk test. For the comparison of nominal variables, a nonparametric Pearson’s Chi-square test was used.

Ordinal-scaled variables (AIS, ISS, GCS, SOFA score) are presented as the median with variance presented as the IQR. Interval-scaled variables (age, BMI, lactate, duration of ventilation, length of ICU stay, length of emergency surgery) are presented as the mean with variance presented as the standard deviation (SD).

Ordinal- and interval-scaled variables were compared using a Mann–Whitney U test. The correlation of significant variables was performed by using a binominal logistic regression analysis. We subdivided the analysis as proposed by Rozenfeld et al. [23] into patients with an ISS between 16 and 24 and patients with an ISS ≥ 25. Mortality was additionally assessed using a Cox hazard regression analysis. The survival rate during in-hospital treatment (in hours) was visualized with a Kaplan–Meier curve. A *p* value < 0.05 was considered statistically significant.

## 3. Results

### 3.1. Demographics

A total of 383 patients were included. The mean age was 51.5 years (±20.4 years) with 71% of patients being male. The most frequent trauma causes (Table 1) were road traffic injuries, followed by falls of a low height (<3 m) and those from a greater height (>3 m).

### 3.2. Status at Admission and Clinical Course

At admission, the median AIS was highest for injuries to the head (median GCS was 7 (IQR 3–15)), followed by the thorax and the extremities (Table 1), while the median ISS was 24 (17–27). The mean admission lactate was 2.92 (±2.65) mmol/L. In total, 205 patients (53.5%) needed emergency surgery on the same day of admission. The mean duration of ICU stay was 13.2 (±21.4) days. A total of 248 patients (64.6%) needed mechanical ventilation, with a mean respiratory time of 290.5 (±483.8) hours (Table 1).

### 3.3. Mortality

**Figure 1:**
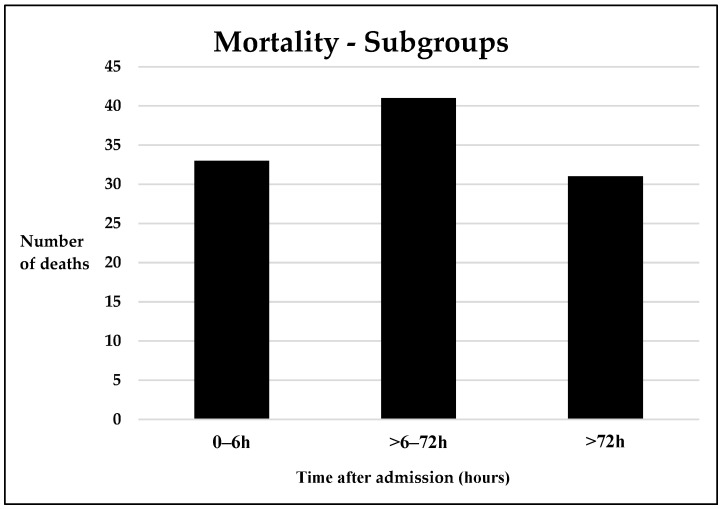
Time-dependent mortality. Number of deaths in each mortality group. h = hours.

In total, 105 patients died within the stationary course (27.4%), with the highest peak between 6 and 72 h (Figure 1). Compared to survivors, deceased patients were significantly older, had a significantly higher median ISS, lower GCS and a higher lactate on admission (Table 2). Compared to survivors, patients that died within three days after admission additionally presented with a significantly higher median SOFA score on admission (Supplementary Table S4). Patients that suffered late posttraumatic mortality had a higher age, more severe head injuries, a lower GCS at admission, lower AIS_extremity_ and higher rates of organ failure (Supplementary Table S4).

#### 3.3.1. Cox Hazard Regression Analysis for In-Hospital Mortality after Trauma

**Figure 2:**
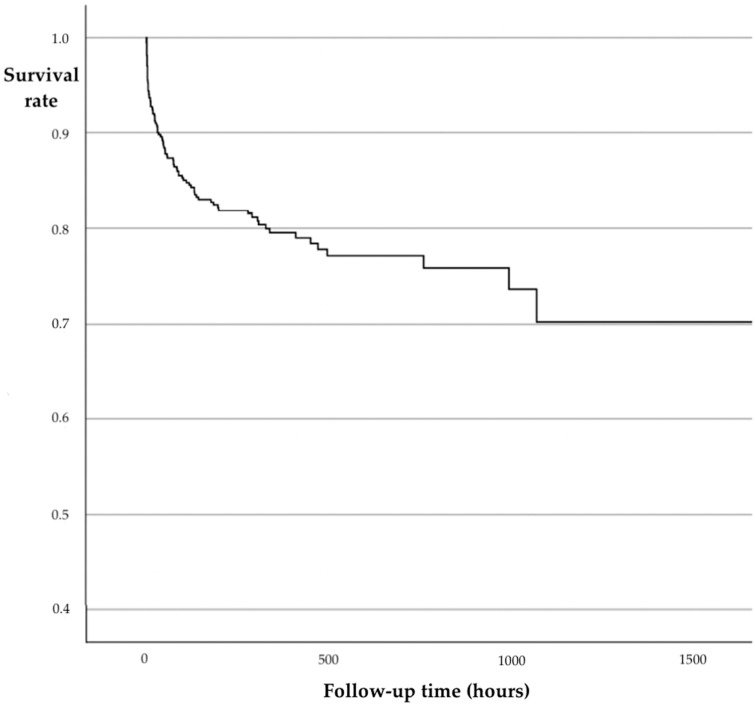
Kaplan–Meier curve for the survival rate in hours after admission. The highest mortality was observed during the first hours and days after the trauma.

For the assessment of potential risk factors that influence mortality during the clinical course, we performed a Cox hazard regression analysis. In this regression analysis including patients from admission to discard or primary endpoint (mortality), an elevated ISS (OR 1.048, 95%-CI 1.028; 1.068, *p* < 0.001), older age (OR 1.027, 95%-CI 1.017; 1.038, *p* < 0.001) but also reduced rates of complications (OR 0.268; 95%-CI 0.172; 0.418, *p* < 0.001) correlated with mortality (Table 3). The data are presented additionally as a Kaplan–Meier curve, highlighting the peak of mortality in the first days after the initial trauma (Figure 2).

#### 3.3.2. Logistic Regression Analysis for In-Hospital Mortality after Trauma

##### Early Mortality

**Figure 3:**
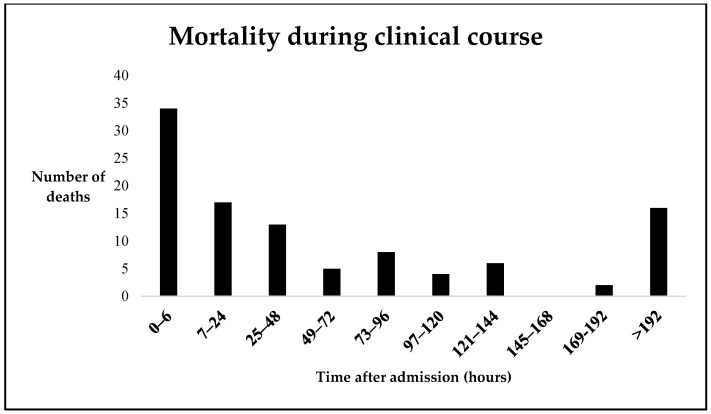
Timeline of mortality within the first 8 days after the trauma. In the first 72 h, the mortality decreases continuously, while after 72 h, there is a second peak in mortality.

To analyze the risk of immediate and early death (0–72 h after admission), a binominal logistic regression was performed. For the logistic regression analysis, we combined immediate and early deaths, as their parameters did not differ significantly in the majority of parameters (despite significant differences in the AIS_Thorax_, lactate at admission and dependent factors ventilation duration and ICU stay, Supplementary Table S4). Also, we considered the continuous decrease in mortality within the first three days, with an additional peak between the third and fifth day (Figure 3).

In the first regression, we included all patients and assessed the risk factors for early or late mortality. The lactate on admission was the only significant factor influencing the risk of death within the first 72 h (OR 1.2, 95%-CI 1.034; 1.433, *p* = 0.018, Table 4). After the subdivision of patients with immediate and early mortality into two separated groups (ISS 16–24 and ISS ≥ 25), no significant factor could be observed in the analysis (Supplementary Tables S1 and S2).

##### Late Mortality

Concerning late death, we used a binominal logistic regression to analyze the effects of the ISS, presence of a severe head or thoracic injury, age or the development of infectious complications. A significant influence on late death compared to the study population, including early deaths, was displayed by the presence of severe head injuries (OR 3.4, 95%-CI 1.218; 10.387; *p* = 0.020, Table 5), age and the development of overall complications (OR 2.7, 95%-CI 1.231; 6.246; *p* = 0.014, Table 5). Due to the significantly reduced rate of complications in the group of patients that died in the first 72 h after admission and the statistical negative correlation with mortality in the Cox hazard regression analysis, we performed an additional logistic regression, excluding patients with immediate and early mortality (Table 6). Compared to survivors, severe head injuries, older age and organ failure (Table 6) were significant, while the overall complication rate had no significant influence on late mortality (Table 6). In patients suffering late mortality with an ISS between 16 and 24, a severe head injury was the only significant factor (OR 7.692, 95%-CI 1.650; 35.852, *p* = 0.009, Supplementary Table S1). Mortality after >72 h in patients with an ISS > 24 correlated significantly with a higher ISS, older age and the development of organ failure (OR 4.291; 95%-CI 1.107; 16.639, *p* = 0.016, Supplementary Table S2).

### 3.4. Complication Rates and Characteristics

A total of 182 (47.5%) patients developed complications, with 100 patients (54.9%) having more than one complication. Overall, 352 complications were observed. In total, 53.7% of all complications were assigned to the “infection cluster”, with pneumonia being the most common complication followed by sepsis (Table 7). The rate of complications was significantly lower in patients with a severe head injury and significantly higher in patients with a severe extremity injury (Supplementary Table S3) and in patients with a higher mean SOFA score at admission (Supplementary Table S3). The complication rates of patients with an immediate or early death were significantly lower (Supplementary Table S4) than in survivors, while in late death, complication rates were higher than in survivors with higher rates in the “infection” cluster and significantly higher in the “organ failure” cluster (Supplementary Table S4).

### 3.5. Complication Cluster Specifics

Patients that developed a complication of the “infection” cluster had significantly higher rates of severe extremity and external injuries, a higher ratio of mechanical ventilation, a longer mean ventilation time and ICU duration and higher rates of emergency operations (Supplementary Table S3).

Patients within the complication cluster “thromboembolism” had a significantly longer ventilation duration and a longer ICU stay (Supplementary Table S3).

Patients with a complication of the “surgery” cluster had less severe head injuries and more severe thoracic and extremity injuries (Supplementary Table S3). Also, in this group, the rate of emergency operations was higher, and the mean duration of the emergency operation was longer (Supplementary Table S3). Patients with complications of the “organ failure” cluster had a higher median SOFA score on admission, longer duration of mechanical ventilation and a longer ICU stay (Supplementary Table S3).

### 3.6. Individual Risk Factors

Significant individual factors influencing the development of complications in general were the ventilation duration, the occurrence of a severe extremity injury (OR 3.359, 95%-CI 1.555; 7.257, *p* = 0.002, Table 8) and the duration of the ICU stay. All three factors were also significant factors for the development of cluster “infection” complications (Table 8). While there were no significant risk factors for cluster “thromboembolism” complications, the risk for complications in the “surgery” cluster was significantly influenced by the presence of a severe thoracic or extremity injury (Table 8). The only significant risk factor influencing the development of “organ failure” cluster complications was the presence of severe thoracic trauma (OR 2.0, 95%-CI 1.085; 3.803, *p* = 0.027, Table 8).

## 4. Discussion

Complications are frequently observed in patients surviving the initial trauma impact. As multiple-trauma patients represent a highly heterogeneous group, the development of complications is hard to predict, and outcomes cannot be well estimated. Therefore, the current study focused on the possible variables that might influence a patient’s clinical course in regard to complications and mortality. The main findings may be summarized as follows: by clustering complications, we were able to assess the specific pathophysiologic groups of complications.

We confirmed the independent influence of severe head injuries, age and organ failure on late mortality. Especially in patients with moderate trauma severity (ISS 16–24), severe head injuries are the main factor influencing late mortality, while patients with a higher injury severity (ISS ≥ 25) are significantly endangered due to the development of organ failure. Interestingly, we observed a significant influence of severe extremity injuries on overall complication rates, infections and surgery-associated complications. The optimal treatment strategy for severe extremity injuries has been in discussion for decades [26]. The elevated risk for complications in patients with severe extremity injuries should be considered when deciding between different surgical treatment options, while further research on the underlying factors could facilitate new treatment options.

Furthermore, the presence of severe thoracic trauma influenced the risk of surgery-associated complications and organ dysfunction. While elevated lactate at admission served as an individual risk factor of early mortality, an early elevated SOFA score was associated with the development of organ failure.

### 4.1. Trauma-Associated Mortality

Despite huge efforts regarding diagnostics and therapy, trauma-related mortality remains high. While the initial trauma impact is responsible for the significant mortality rates at the scene of an accident or injury, so-called late death is still responsible for 22% of the mortality of all hospitalized trauma patients [1]. Data from this analysis present an overall in-hospital mortality rate of 27.4%, which is among the upper range according to a recent meta-analysis of comparable studies [8]. This might be explained by the inclusion criteria: in contrast to other authors or data registries that exclude patients who died during the initial trauma management, this study included all severely injured patients after hospital admission. Our study also included patients that were admitted under resuscitation and patients with very high trauma severities. These patients are especially endangered by immediate mortality, thus increasing the mortality rate tremendously. When excluding deaths within 48 h after admission, the mortality rate decreases to 11% and is comparable to the reported in-hospital mortality after 48 h by van Wessel et al. (17%) [27] or Ciesla et al. (8%) [28].

Trauma-associated mortality was classically described by the “trimodal” mortality distribution, with a first peak within the first hour after the trauma and a second early peak within the first four hours. A third peak was described by Trunkey one week after the trauma [29]. More recent studies have confirmed the first and second early peak, while a third peak was not observed. The authors concluded that the third peak was not observed due to improved trauma system implementation and reduced rates of MODS [30]. In our analysis, we were able to observe a trend toward a third increase in mortality between the third and fifth day after the initial trauma (Figure 3), within an overall continuous decrease in mortality (Figure 2). The Cox hazard regression analysis for overall mortality showed an older age and a higher injury severity are significant factors for mortality. This is in line with previously described data [10,31]. These findings underline the previously stated demand for improved injury prevention and road traffic safety measures to reduce injury severity and trauma-related mortality [32].

Interestingly, in the hazard analysis, the development of complications negatively correlated with the overall mortality. This can be explained by the low rates of included complications (Supplementary Table S4) in the groups of immediate (6.1%) and early mortality (19.5%) compared to survivors (54.9%) and patients with late mortality (64.5%). As demonstrated by the early peak of mortality, most patients included in this study died within 72 h after admission, which can be explained by the severity of the sustained injuries, quantified by ISS, and their increased age, which has been previously described [10]. Due to the high impact of the initial trauma leading to early mortality, patients died before the included complications could develop, thus explaining the low complication rates in the early mortality groups. Infection, as the leading complication (Table 7), has been described to usually develop not until several days after multiple trauma [33].

The regression analysis performed in the presented study proved an elevated lactate level on admission as an independent risk factor for immediate and early mortality. As other authors have described a correlation between injury severity and lactate levels before, the present study confirms the validity of this marker in regard to early mortality in multiple-trauma patients [34,35,36]. However, an elevated lactate level has to be interpreted carefully, as other factors (e.g., alcohol intoxication [37]) can impact the diagnostic value. Thus, lactate should only be used for the evaluation of polytraumatized patients in combination with other clinical and blood markers (including physiological parameters and coagulopathy) [13].

Regarding late mortality, patients were older, displayed more severe head injuries and developed more complications (overall, clusters “infection” and “organ failure”) compared to patients with immediate and early mortality (Supplementary Table S4).

To avoid the potential bias concerning the low rates of complications in the early mortality groups, we compared the influence of mortality in late deaths with survivors only. Compared to a significant influence of complications on late mortality including all patients (Table 5), by excluding patients with immediate and early mortality, the impact of complication development was not significant (Table 6).

Late mortality was influenced significantly by older age, a severe head injury, and the development of organ failure (Table 6). The increasing importance of a head injury in patients with multiple trauma and poorer outcomes has been described previously [8]. For patients with a moderate ISS between 16 and 24, a severe head injury was the only significant factor influencing late in-hospital mortality (OR 7.692, 95%-CI 1.650; 35.852, *p* = 0.009, Supplementary Table S1). The increasing impact of traumatic brain injuries has been demonstrated by a recent meta-analysis [8] and can be explained by the ongoing limited therapeutic options for traumatic brain injuries. The current options include strategies to prevent any secondary damage to the brain after the primary injury, while there are no therapeutic options for the primary damage [38]. Complications after a traumatic brain injury include, among others, edema, increased intracranial pressure, oxidative stress and coagulopathy [39] that can lead to posttraumatic mortality [40]. As our study shows, especially patients with a moderate trauma severity and severe head trauma are at high risk of late trauma-associated mortality.

In our study population, late mortality in patients with a high injury severity (ISS ≥ 25) correlated significantly with a higher age and the development of organ failure (Supplementary Table S2). Although the attributed mortality to organ failure after trauma has been reported to decrease [41], we showed for patients with an ISS ≥ 25 that organ failure is still a complication significantly influencing late mortality.

### 4.2. The Development of Posttraumatic Complications

Multiple-trauma patients that survive the impact of the initial trauma are highly endangered to developing complications, negatively influencing the further clinical course. Although the included complications in our study did not significantly correlate with late mortality, the high rates of complications in survivors and patients with late mortality emphasize the tremendous impact of trauma severity on the clinical course.

While several authors have reported complication rates of up to 12.7% in less injured patients [42,43], data from the present study proved higher rates in severely injured patients. Infections were observed most often (in 35.5% of all included patients, Table 7). A comparable study by Halvachizadeh et al., with a study population of multiple-trauma patients with an ISS ≥ 16, reported similar in-hospital rates of pneumonia (19%) and sepsis (14.9%) [44].

Within our study population, severe extremity injuries correlated positively (OR 3.4, 95%-CI 1.555; 7.257, *p* = 0.002, Table 8) with the development of complications. A similar association was described by Poole et al., with higher rates of ARDS and longer ICU stays in patients with more severe extremity injuries independent of the ISS [45]. Zeelenberg et al. described a positive correlation between a higher AIS of the upper extremity and a longer ICU stay in polytraumatized patients [46]. A severe extremity injury can be associated with significant blood loss, thus leading to immunological changes [47] which potentially influence the susceptibility to infection. These changes can especially be relevant in patients with open extremity injuries. Several studies have shown how the immune system is dysregulated by an additional severe hemorrhage [48,49,50]. Despite consecutive changes regarding the immunologic response, Poole et al. assume systemic hypoperfusion as well as severe soft tissue trauma to contribute negatively to the development of complications (e.g., infection). Furthermore, they conclude that a higher trauma impact and severe extremity injuries are associated with severe concomitant injuries of other body regions, increasing the vulnerability to complications [45]. Additionally, severe extremity injuries potentially impede early posttraumatic mobilization, raising the risk for the most observed complication, pneumonia [51]. Especially severe injuries of the upper extremity in multiple-injury patients are associated with a higher complexity and impeded functional recovery [52]. However, the definitive impact of extremity injury severity on infections remains elusive.

The influence of severe thoracic trauma on the development of infections in multiple-trauma patients is an ongoing discussion. While Seibold et al. described thoracic trauma contributed to pulmonal organ damage by locally active inflammatory mediators in an animal model [53], the clinical data are inconclusive. In smaller study populations, no impact of severe thoracic trauma on complication rates was described [6], whereas larger studies show a significant higher rate of sepsis, lung failure [54] or mortality [5]. In our analysis, severe thoracic trauma was a significant factor influencing the occurrence of organ failure, including acute renal failure and ARDS (OR 2.0, 95%-CI 1.085; 3.803, *p* = 0.027, Table 8). Thus, the presented data support the findings of Seibold et al. regarding remote organ damage correlating with an increased risk of late posttraumatic mortality. Despite the impact of thoracic trauma on the immune system, other factors could also increase the susceptibility to infection and concomitant respiratory failure. For patients with severe thoracic trauma, longer durations of mechanical ventilation are described [54], which is a known risk factor for pneumonia in polytraumatized patients [55]. Also, thoracic trauma is, in many cases, associated with severe and prolonged pain, impeding deep pulmonal ventilation, causing hypoventilation, atelectasis and pneumonia [56].

Additional to organ failure, severe thoracic trauma (OR 2.5, 95%-CI 1.136; 5.405, *p* = 0.023, Table 8) but also extremity injuries (OR 3.3, 95%-CI 1.487; 7.234, *p* = 0.003, Table 8) significantly contributed to the development of surgical complications. While complications like wound-healing disorders might be triggered by impaired microperfusion or local inflammatory changes on the one hand [57], the management of complex extremity injures and advanced surgical demands might be responsible for complications like wound-healing disorders on the other [58]. This is underlined by an observed significant longer mean operation time during emergency surgery in patients with surgical complications (Supplementary Table S3).

Regarding infection, the duration of mechanical ventilation and a prolonged ICU stay was shown to have a significant influence. Thus, the presented data confirm the previously published findings reporting a positive correlation between mechanical ventilation and the development of pneumonia in polytraumatized patients [59]. Although mechanical ventilation is a known risk factor for pneumonia in general [60], additional injury-related thoracic and pulmonal damage does have an aggravating effect via local tissue damage, histological changes and immunological processes that might increase the risk of infection in polytraumatized patients [61]. While the impact of multiple trauma on the immune system [62,63,64] and the resulting elevated susceptibility to pneumonia [65,66] have been described in several in vitro and animal models, our study focused on clinical parameters. Future studies focusing on the direct correlation between infections and immunological disbalances are needed.

## 5. Strengths and Limitations

Aside from the considerable number of patients and detailed information gained from the hospital information system, the retrospective character of this study must be named as a limitation. Moreover, polytraumatized patients are characterized by a very heterogenous injury pattern that calls for individual treatment strategies. This, in turn, might cause some bias regarding the development of complications. As this study focused on trauma-associated in-hospital mortality, we did not include pre-hospital mortality. Regarding overall trauma-associated mortality and the distribution of mortality, this must be considered carefully. Furthermore, complications were assigned to clusters out of pathophysiologic considerations or therapeutic strategies (e.g., compartment syndrome). These allocations must be considered when interpreting the data. The patients that were included in this study were treated between 2010 and 2014, thus the interpretation of the data must consider the treating standards in these years, considering changes in the treatment guidelines and strategies until publication. Finally, males are the predominant trauma group. A gender specific subanalysis was not performed due to the low number of women affected by severe trauma. We emphasize to carefully consider the gender differences regarding our data that should be evaluated in larger study populations.

## 6. Conclusions

Trauma severity significantly influences early (0–72 h after admission) mortality in polytraumatized patients. Late mortality in patients with moderate trauma severity (ISS 16–24) is mainly influenced by severe head injuries. In patients with an ISS ≥ 25, the development of organ failure leads to late mortality. Overall, complication rates among polytraumatized patients remain high, with elevated rates of infections and organ failure. Special care should be given to patients with severe extremity injuries, a severe head injury and patients needing prolonged mechanical ventilation. These patients are at an elevated risk of developing complications during the clinical course. Further efforts should be made to understand the underlying mechanisms and reduce complication rates. Furthermore, the importance of trauma prevention systems and improved road traffic safety, focusing on head injuries, is underlined by the significant influence of trauma severity on trauma-associated mortality.

## Figures and Tables

**Table 1:** General demographic parameters. Data are presented as the mean (±standard deviation (SD) = *) for the interval-scaled variables. Ordinal-scaled values are presented as the median (±interquartile range (IQR) = #). BMI = body mass index, AIS = abbreviated injury scale, ISS = injury severity score, SOFA = sequential organ failure assessment, GCS = Glasgow Coma Scale, ICU = intensive care unit, h = hours, d = days.

Parameters		Mean/Median (±SD/IQR)
Epidemiology	Age (years)	51.5 (±20.4 *)
	BMI	25.9 (±4.1 *)
	Gender (% male)	71%
Injury mechanism	Road traffic car (n; %)	60 (15.7)
	Road traffic motorcycle (n; %)	45 (11.7)
	Road traffic bike (n; %)	27 (7)
	Road traffic pedestrian (n; %)	34 (8.9)
	Fall > 3 m height (n; %)	61 (15.9)
	Fall < 3 m height (n; %)	92 (24)
	Explosion trauma (n; %)	10 (2.6)
	Others (n; %)	54 (14.1)
Trauma severity (median)	ISS	24 (17–27 #)
	AIS head	3 (0–4 #))
	AIS face	0 (0–0 #)
	AIS thorax	2 (0–3 #)
	AIS abdomen	0 (0–2 #)
	AIS extremities	2 (0–3 #)
	AIS external	0 (0–1 #)
Parameters on admission	GCS	7 (3–15 #)
	SOFA	6 (4–8 #)
	Lactate (mmol/L)	2.92 (±2.65 *)
Onset parameters	Duration of ventilation (h)	290.5 (±483.8 *)
	ICU stay (d)	13.2 (±21.4 *)
	Length of emergency surgery (min)	146.8 (±116.3 *)
	Mortality (n; %)	106 (27.7)

**Table 2:** Factors of different time-dependent mortality. *p* values compare overall mortality versus survivors. Data are presented as the mean (±standard deviation (SD)) for the interval-scaled variables. Ordinal-scaled values are presented as the median (±interquartile range (IQR)). BMI = body mass index, AIS = abbreviated injury scale, ISS = injury severity score, SOFA = sequential organ failure assessment, GCS = Glasgow Coma Scale, ICU = intensive care unit, h = hours, d = days.

Parameter	Death Overall (n = 106)			Death Immediate	Death Early	Death Late
	Yes	No	*p*			
Age (years)	61.0	47.9	<0.001	59.5	58.3	66.1
SD	21.1	18.9		20.7	23.9	19.2
BMI	26.1	25.9	0.751	25.8	26.7	25.4
SD	3.9	4.2		3.2	4.0	4.0
Gender (% male)	67.0	72.6	0.272	60.6	70.7	67.7
AIS head (% severe)	77.4	51.6	<0.001	66.7	78.0	83.9
AIS face (% severe)	7.5	9.4	0.571	3.0	14.6	3.2
AIS abdomen (% severe)	12.3	14.8	0.523	21.2	14.6	3.2
AIS thorax (% severe)	35.8	45.5	0.088	51.5	24.4	35.5
AIS extremity (% severe)	22.6	30.3	0.135	30.3	22.0	12.9
AIS external (% severe)	5.7	2.5	0.130	6.1	2.4	9.7
ISS	25	22	<0.001	26	25	25
IQR	20–30	17–27		23–34	24–29	18–27
SOFA	7	5	<0.001	7	8	6
IQR	6–9	4–8		6–8	7–9	4–8
GCS	3	11	<0.001	3	3	3
IQR	3–9	3–15		3–4	3–9	3–11
Lactate (mmol/L)	4.7	2.3	<0.001	6.8	3.7	3.9
SD	4.0	1.5		4.7	2.6	3.5
Ventilation duration (h)	138	357	<0.001	3	32	337
SD	302.0	532.2		1.4	22.9	434.2
ICU stay (d)	5.7	16	<0.001	1	2	15
SD	12.2	23.4		0.4	1.1	19.7
Emergency operation duration (min)	126	153	0.143	54.0	135.9	140
SD	97.5	120.8		30.5	98.5	95.9
Complication rate (%)	28.3	54.9	<0.001	6.1	19.5	64.5
Cluster Infection (%)	17.0	42.6	<0.001	0.0	2.4	54.8
Cluster Thromboemolism (%)	2.8	6.5	0.16	0.0	0.0	9.7
Cluster Surgery (%)	6.6	15.9	0.017	0.0	2.4	19.4
Cluster Organ failure (%)	19.8	19.5	0.944	6.1	17.1	38.7

**Table 3:** Cox hazard regression analysis for mortality. Severe injuries are defined as AIS > 2 in the distinct region. ISS = injury severity score, OR = Odds ratio, CI = confidence interval.

Mortality	ISS	Severe Head Injury	Severe Thorax Injury	Age	Complication
OR	1.048	1.664	0.751	1.027	0.268
95%-CI	1.028; 1.068	0.994; 2.783	0.474; 1.188	1.017; 1.038	0.172; 0.418
*p*	<0.001	0.053	0.221	<0.001	<0.001

**Table 4:** Odds ratio of time-dependent mortality, including early (0–72 h) mortality compared to all included patients. Severe injuries are defined as an AIS > 2 in the distinct region. ISS = injury severity score, OR = odds ratio, CI = confidence interval.

Mortality Early	ISS	Severe Head Injury	Age	Lactate	SOFA Score
OR	1.043	1.367	1.001	1.217	1.196
95%-CI	1.000; 1.088	0.503; 3.720	0.978; 1.025	1.034; 1.433	0.976; 1.466
*p*	0.052	0.540	0.921	0.018	0.085

**Table 5:** Odds ratio of time-dependent mortality, including late (>72 h after admission) mortality compared to all included patients. Severe injuries are defined as an AIS > 2 in the distinct region. ISS = injury severity score, OR = odds ratio, CI = confidence interval.

Mortality Late	ISS	Severe Head Injury	Severe Thorax Injury	Age	Complication
OR	1.028	3.557	0.998	1.041	2.773
95%-CI	0.987; 1.071	1.218; 10.387	0.401; 2.482	1.019; 1.064	1.231; 6.246
*p*	0.128	0.020	0.997	<0.001	0.014

**Table 6:** Odds ratio of time-dependent mortality, including late (>72 h after admission) mortality compared to survivors only. Severe injuries are defined as an AIS > 2 in the distinct region. ISS = injury severity score, OR = odds ratio, CI = confidence interval.

Mortality Late	ISS	Severe Head Injury	Severe Thorax Injury	Age	Complication
OR	1.039	3.326	0.944	1.050	1.364
95%-CI	0.995; 1.085	1.114; 9.929	0.369; 2.414	1.025; 1.075	0.592; 3.146
*p*	0.081	0.031	0.904	<0.001	0.466
**Mortality Late**	**ISS**	**Severe head injury**	**Age**	**Organ failure**	
OR	1.039	3.485	1.050	2.419	
95%-CI	0.997; 1.082	1.242; 9.780	1.025; 1.076	1.040; 5.630	
*p*	0.067	0.018	<0.001	0.040	

**Table 7:** Included complications and their cluster allocation. Severe injuries are defined as an AIS > 2 in the distinct region. ISS = injury severity score, OR = odds ratio, CI = confidence interval.

Cluster of Complication	Complication	Quantity (% of All Patients; % of All Complications)
Infection	Pneumonia	83 (21.7%; 23.6%)
	Urinary tract infection	19 (5%; 5.4%)
	Wound infection	34 (8.9%; 9.7%)
	Sepsis [22]	53 (13.8%; 15.1%)
Thromboembolism	Myocardial infarction	2 (0.5%; 0.6%)
	Brain infarction	8 (2.1%; 2.3%)
	Thrombosis	7 (1.8%; 2%)
	Pulmonary embolism	5 (1.3%; 1.4%)
Surgical treatment associated (Surgery)	Compartment syndrome	5 (1.3%; 1.4%)
	Hematoma/Seroma	8 (2.1%; 2.3%)
	Wound-healing disorders	12 (3.1%; 3.4%)
	Nerve damage	20 (5.2%; 5.7%)
	Implant-associated complications	13 (3.4%; 3.7%)
Organ failure	Acute respiratory distress syndrome (ARDS) [24]	51 (13.3%; 14.5%)
	Acute renal failure [25]	32 (8.5%; 9.1%)

**Table 8:** Odds ratio (OR) of patient or injury characteristics on the development of complications. Severe injuries are defined as an AIS > 2 in the distinct region. ISS = injury severity score, OR = odds ratio, CI = confidence interval, ICU = intensive care unit.

Complication		ISS	Age	Severe Thorax Injury	Severe Extremity Injury	Severe External Injury	Mechanical Ventilation Duration	ICU Stay
Overall	OR	0.987	1.008	1.754	3.359	4.028	1.002	1.061
	95%-CI	0.952; 1.023	0.993; 1.023	0.924; 3.331	1.555; 7.257	0.597; 27.156	1.000; 1.005	1.014; 1.111
	*p*	0.462	0.291	0.086	0.002	0.152	0.038	0.010
Cluster Infection	OR	0.992	1.010	0.928	2.947	2.965	1.004	1.047
	95%-CI	0.956; 1.030	0.994; 1.025	0.531; 2.004	1.278; 6.303	0.447; 19.657	1.001; 1.006	1.003; 1.093
	*p*	0.677	0.220	0.928	0.005	0.260	0.002	0.037
Cluster Thrombo-embolism	OR	0.998	0.996	1.223	0.918	0.000	1.003	0.976
	95%-CI	0.931; 1.069	0.970; 1.021	0.398; 3.756	0.257; 3.284	0.000	1.000; 1.006	0.925; 1.030
	*p*	0.950	0.732	0.725	0.895	0.999	0.054	0.375
Cluster Surgery	OR	0.986	0.996	2.478	3.280	0.000	1.001	1.004
	95%-CI	0.942; 1.032	0.978; 1.015	1.136; 5.405	1.487; 7.234	0.000	0.999; 1.003	0.960; 1.051
	*p*	0.549	0.693	0.023	0.003	0.999	0.456	0.849
Cluster Organ failure	OR	0.980	1.013	2.031	1.522	3.710	1.001	0.996
	95%-CI	0.946; 1.014	0.998; 1.028	1.085; 3.803	0.751; 3.087	0.875; 15.726	0.999; 1.003	0.961; 1.033
	*p*	0.246	0.095	0.027	0.244	0.075	0.178	0.835

## Data Availability

Additional data (e.g., raw data) will be provided upon reasonable request. Minor parts of this study have been published as meeting abstracts.

## Supplementary Materials

The following supporting information can be downloaded at: https://www.mdpi.com/article/10.3390/jcm12155111/s1, Table S1: Odds ratio (OR) of time-dependent mortality in patients with ISS 16-24. Late mortality was assessed vs. survivors only; Table S2: Odds ratio (OR) of time-dependent mortality in patients with ISS ≥ 25. Late mortality was assessed vs. survivors only; Table S3: Factors of complication development. Significance values are presented for patients with a complication (or distinct complication) vs. patients with no complication in the distinct cluster (or overall); Table S4: Factors of mortality development. Significance values are presented for patients with immediate, early or late mortality vs. survivors or patients with early mortality.
