# Supplementary material for: Effect of Injury Patterns on the Development of Complications and Trauma-Induced Mortality in Patients Suffering Multiple Trauma
Source: J Clin Med. 2023 Aug 3;12(15):5111. doi: 10.3390/jcm12155111 (PMC10420136; doi:10.3390/jcm12155111)
Supplement: Supplementary file 1 [file jcm-12-05111-s001.zip › jcm-2508168-supplementary.pdf]

Supplementary Material

Supplementary Table 1. Odds ratio (OR) of time-dependent mortality in patients with ISS 16-24. Late mortality was assessed vs. survivors only. Severe injuries are defined as AIS > 2 in the distinct region. ISS = injury severity score, OR = Odds ratio, CI = confidence interval, SOFA = Sequential organ failure assessment score.

**Mortality early**	**ISS**	**Severe head injury**	**Age**	**Lactate**	**SOFA Score**
**OR**	**0.995**	**0.477**	**1.064**	**1.429**	**1.119**
95%- CI	0.574; 1.722	0.503; 5.111	0.986; 1.148	0.983; 2.077	0.736; 1.703
p	0.985	0.395	0.110	0.062	0.599
**Mortality late**	**ISS**	**Severe head injury**	**Age**	**Organ failure**	
**OR**	**0.978**	**7.692**	**1.024**	**1.859**	
95%- CI	0.794; 1.205	1.650; 35.852	0.992; 1.057	0.551; 6.272	
p	0.836	0.009	0.143	0.317	

Supplementary Table 2. Odds ratio (OR) of time-dependent mortality in patients with ISS ≥ 25. Late mortality was assessed vs. survivors only. Severe injuries are defined as AIS > 2 in the distinct region. ISS = injury severity score, OR = Odds ratio, CI = confidence interval. SOFA = Sequential organ failure assessment score.

**Mortality early**	**ISS**	**Severe head injury**	**Age**	**Lactate**	**SOFA Score**
**OR**	**1.004**	**1.275**	**0.988**	**1.231**	**1.128**
95%- CI	0.946; 1.066	0.383; 4.250	0.961; 1.016	0.961; 1.578	0.888; 1.434
p	0.887	0.692	0.390	0.100	0.323
**Mortality late**	**ISS**	**Severe head injury**	**Age**	**Cluster Organ failure**	
**OR**	**1.067**	**1.710**	**1.091**	**4.291**	
95%- CI	1.004; 1.134	0.334; 8.748	1.043; 1.142	1.107; 16.639	
p	0.035	0.529	< 0.001	0.016	

Supplementary Table 3. Factors of complication development. Significance values are presented for patients with a complication (or distinct complication) vs. patients with no complication in the distinct cluster (or overall). Severe injuries are defined as AIS > 2 in the distinct region. ISS = injury severity score, OR = Odds ratio, CI = confidence interval.

**Parameter**	**Complication Overall** *(n=182)*			**Cluster Infection** *(n=136)*			**Cluster Thrombo-embolism** *(n=21)*			**Cluster Surgery** *(n=51)*			**Cluster Organ failure** *(n=73)*		
	**Yes**	**No**	**p**	**Yes**	**No**	**p**	**Yes**	**No**	**p**	**Yes**	**No**	**p**	**Yes**	**No**	**p**
Age (years)	51.5	51.5	0.979	52.7	50.1	0.403	51.6	51.5	0.960	47.0	52.2	0.095	54.2	50.8	0.203
SD	*20.5*	*20.5*		*20.6*	*20.5*		*17.6*	*20.7*		*19.2*	*20.6*		*18.8*	*20.9*	
BMI	25.8	26.0	0.692	25.7	26.1	0.464	25.8	25.9	0.791	25.8	25.9	0.698	26.4	25.8	0.108
SD	*3.8*	*4.3*		*3.9*	*4.2*		*3.3*	*4.1*		*4.1*	*4.1*		*4.0*	*4.1*	
Gender (%male)	68.7	73.1	0.337	69.1	72.1	0.543	81.0	70.4	0.302	64.7	72.0	0.286	74.7	70.1	0.437
AIS head (% severe)	52.7	64.7	0.018	55.1	61.1	0.254	61.9	58.8	0.781	40.4	61.7	0.005	54.7	60.1	0.394
AIS face (% severe)	7.7	10.0	0.438	8.8	8.9	0.978	9.5	8.8	0.915	9.8	8.7	0.803	5.3	9.7	0.229
AIS abdomen (% severe)	15.9	12.4	0.326	14.7	13.8	0.800	9.5	14.4	0.535	19.6	13.3	0.225	14.7	14.0	0.875
AIS thorax (% severe)	46.2	39.8	0.210	42.6	42.9	0.960	47.6	42.5	0.648	62.7	39.8	0.002	50.7	40.9	0.126
AIS extremity (% severe)	35.2	21.9	0.004	35.3	24.3	0.022	28.6	28.2	0.969	51.0	24.7	< 0.001	33.3	26.9	0.270
AIS external (% severe)	4.9	2.0	0.111	5.9	2.0	0.046	0.0	3.6	0.377	0.0	3.9	0.151	6.7	2.6	0.081
ISS	22	24	0.726	22	24	0.594	22	24	0.824	22	24	0.371	24	22	0.382
IQR	*17-29*	*18-26*		*17-29*	*18-27*		*17-29*	*17-27*		*18-29*	*17-27*		*17-29*	*17-27*	
SOFA	6	5	0.015	7	5	0.016	7	6	0.239	5	6	0.458	8	5	< 0.001
IQR	*4-8*	*4-7*		*4-8*	*4-8*		*5-8*	*4-8*		*3-8*	*4-8*		*5-9*	*4-7*	
GCS	7	6	0.411	6	7	0.515	3	7	0.085	7	7	0.854	3	8	< 0.001
IQR	*3-15*	*3-15*		*3-15*	*3-15*		*3-10*	*3-15*		*3-15*	*3-15*		*3-13*	*3-15*	
Lactate (mmol/l)	2.6	3.2	0.644	2.7	3.0	0.886	2.48	2.94	0.889	3.1	2.9	0.766	3.14	2.86	0.287
SD	*2.0*	*3.1*		*2.1*	*2.9*		*1.5*	*2.7*		*2.8*	*2.6*		*2.5*	*2.7*	
Ventilation duration (h)	420	111	< 0.001	501	121	< 0.001	857	248	< 0.001	518	245	< 0.001	462	224	< 0.001
SD	*576.9*	*207.0*		*629.6*	*201.1*		*1285.6*	*328.8*		*872.6*	*344.6*		*720.4*	*331.7*	
ICU stay (d)	21	6	< 0.001	25	7	< 0.001	38	12	< 0.001	26	11	< 0.001	25	10	< 0.001
SD	*27.6*	*8.5*		*30.6*	*8.9*		*60.1*	*15.7*		*38.7*	*16.6*		*35.5*	*15.0*	
Emergency operation duration (min)	162	128	0.037	166	133	0.140	150	147	0.530	160	144	0.021	146	147	0.714
SD	*127.1*	*98.9*		*137.1*	*96.6*		*167.0*	*112.9*		*81.6*	*123.2*		*110.0*	*118.4*	

Death overall	16.5	37.8	< 0.001	13.2	35.6	< 0.001	14.3	28.5	0.158	13.7	29.8	0.017	28.0	27.6	0.932
Immediate death (%)	1.1	15.4	< 0.001	0.0	13.4	< 0.001	0.0	9.1	0.158	0.0	9.9	0.019	2.7	10.1	0.041
Early death (%)	4.4	16.4	< 0.001	0.7	16.2	< 0.001	0.0	11.3	0.103	2.0	12.0	0.030	9.3	11.0	0.668
Late death (%)	11.0	5.5	0.048	12.5	5.7	0.019	14.3	7.7	0.285	11.8	7.5	0.302	16	6.2	0.005

Supplementary Table 4. Factors of mortality development. Significance values are presented for patients with immediate, early or late mortality vs. survivors or patients with early mortality. Severe injuries are defined as AIS > 2 in the distinct region. ISS = injury severity score, OR = Odds ratio, CI = confidence interval.

**Parameter**	**Death overall** *(n=106)*			**Death immediate**	**Vs. survivors**	**Vs. early**	**Death early**	**Vs. survivors**	**Death late**	**Vs. survivors**
	**Yes**	**No**	**p**		**p**	**p**		**p**		**P**
Age (years)	61.0	47.9	< 0.001	59.5	<0.001	0.773	58.3	0.017	66.1	<0.001
SD	*21.1*	*18.9*		*20.7*			*23.9*		*19.2*	
BMI	26.1	25.9	0.751	25.8	0.198	0.465	26.7	0.614	25.4	0.271
SD	*3.9*	*4.2*		*3.2*			*4.0*		*4.0*	
Gender (% male)	67.0	72.6	0.272	60.6	0.189	0.425	70.7	0.845	67.7	0.570
AIS head (% severe)	77.4	51.6	< 0.001	66.7	0.016	0.290	78.0	0.005	83.9	<0.001
AIS face (% severe)	7.5	9.4	0.571	3.0	0.801	0.057	14.6	0.822	3.2	0.250
AIS abdomen (% severe)	12.3	14.8	0.523	21.2	0.513	0.059	14.6	0.788	3.2	0.075
AIS thorax (% severe)	35.8	45.5	0.088	51.5	0.797	0.003	24.4	0.054	35.5	0.288
AIS extremity (% severe)	22.6	30.3	0.135	30.3	0.941	0.502	22.0	0.400	12.9	0.042
AIS external (% severe)	5.7	2.5	0.130	6.1	0.324	0.537	2.4	0.969	9.7	0.033
ISS	25	22	< 0.001	26	0.003	0.681	25	<0.001	25	0.346
IQR	*20-30*	*17-27*		*23-34*			*24-29*		*18-27*	
SOFA	7	5	< 0.001	7	0.002	0.807	8	0.002	6	0.174
IQR	*6-9*	*4-8*		*6-8*			*7-9*		*4-8*	
GCS	3	11	< 0.001	3	<0.001	0.990	3	<0.001	3	0.022
IQR	*3-9*	*3-15*		*3-4*			*3-9*		*3-11*	
Lactate (mmol/l)	4.7	2.3	< 0.001	6.8	<0.001	0.024	3.7	0.097	3.9	0.093
SD	*4.0*	*1.5*		*4.7*			*2.6*		*3.5*	
Ventilation duration (h)	138	357	< 0.001	3	<0.001	<0.001	32	<0.001	337	0.820
SD	*302.0*	*532.2*		*1.4*			*22.9*		*434.2*	
ICU stay (d)	5.7	16	< 0.001	1	<0.001	<0.001	2	<0.001	15	0.749
SD	*12.2*	*23.4*		*0.4*			*1.1*		*19.7*	
Emergency operation duration (min)	126	153	0.143	54.0	0.253	0.597	135.9	0.134	140	0.898
SD	*97.5*	*120.8*		*30.5*			*98.5*		*95.9*	
Complication Rate (%)	28.3	54.9	< 0.001	6.1	<0.001	0.059	19.5	<0.001	64.5	0.305
Cluster Infection (%)	17.0	42.6	< 0.001	0.0	<0.001	0.443	2.4	<0.001	54.8	0.193
Cluster Thromboemolism (%)	2.8	6.5	0.16	0.0	0.110	-	0.0	0.106	9.7	0.505
Cluster Surgery (%)	6.6	15.9	0.017	0.0	0.009	0.443	2.4	0.029	19.4	0.619
Cluster Organ-failure (%)	19.8	19.5	0.944	6.1	0.091	0.126	17.1	0.585	38.7	0.013
